# Two-Stage Alignment of FIB-SEM Images of Rock Samples

**DOI:** 10.3390/jimaging6100107

**Published:** 2020-10-10

**Authors:** Iryna Reimers, Ilia Safonov, Anton Kornilov, Ivan Yakimchuk

**Affiliations:** 1 Schlumberger Moscow Research Center, 125171 Moscow, Russia; akornilov@slb.com (A.K.); iyakimchuk@slb.com (I.Y.); 2 Computer Science and Computational Mathematics Department, Moscow Institute of Physics and Technology, 141701 Moscow, Russia; 3 Computer Science and Control Systems, National Research Nuclear University, MEPhI, 115409 Moscow, Russia

**Keywords:** FIB-SEM, alignment, digital rock, stack of images, porous media

## Abstract

Focused Ion Beam Scanning Electron Microscopy (FIB-SEM) tomography provides a stack of images that represent serial slices of the sample. These images are displaced relatively to each other, and an alignment procedure is required. Traditional methods for alignment of a 3D image are based on a comparison of two adjacent slices. However, such algorithms are easily confused by anisotropy in the sample structure or even experiment geometry in the case of porous media. This may lead to significant distortions in the pore space geometry, if there are no stable fiducial marks in the frame. In this paper, we propose a new method, which meaningfully extends existing alignment procedures. Our technique allows the correction of random misalignments between slices and, at the same time, preserves the overall geometrical structure of the specimen. We consider displacements produced by existing alignment algorithms as a signal and decompose it into low and high-frequency components. Final transformations exclude slow variations and contain only high frequency variations that represent random shifts that need to be corrected. The proposed algorithm can operate with not only translations but also with arbitrary affine transformations. We demonstrate the performance of our approach on a synthetic dataset and two real FIB-SEM images of natural rock.

## 1. Introduction

**Figure 1:**
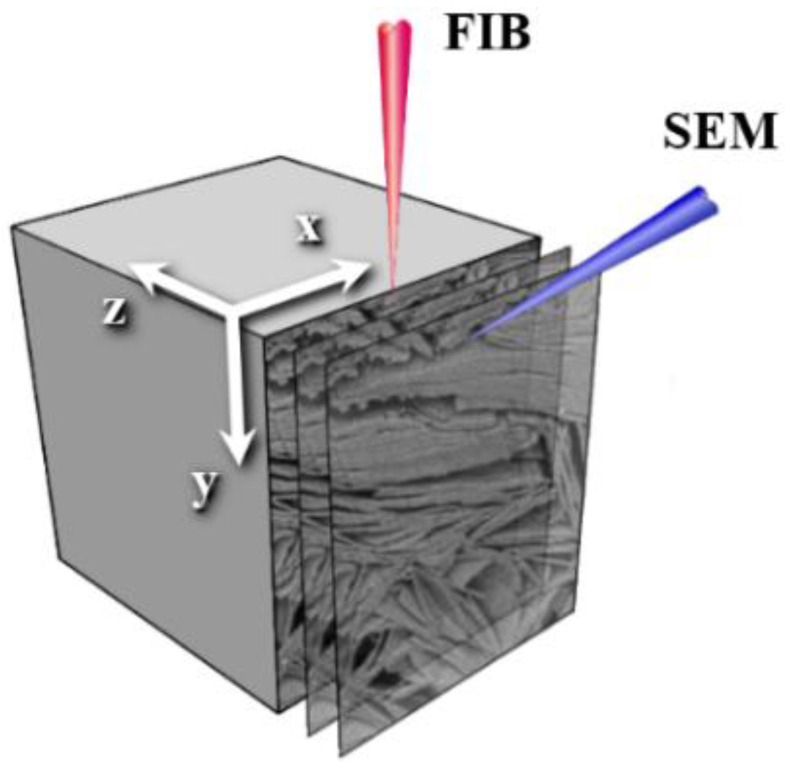
A scheme of Focused Ion Beam Scanning Electron Microscopy (FIB-SEM) tomography.

Focused Ion Beam Scanning Electron Microscopy (FIB-SEM) is a powerful technique for 3D serial imaging at the nanoscale. In the FIB column, there is a source of ions, which are then accelerated and focused into the beam. Striking the sample, they sputter atoms from the surface and, in such a way, etch the specimen. After the ion beam removes a thin layer of substance from the sample, the electron microscope scans the surface at an angle that usually equals 52 degrees. Multiple repetitions of these two operations produce a stack of images that correspond to serial slices of a sample. Figure 1 illustrates the FIB-SEM image acquisition procedure. This technology makes it possible to to obtain substantially higher resolution (5–10 nm) compared to, for example, X-ray tomography (about 2–4 μm), so it is widely used in various fields of science, such as investigation of fuel cells electrodes, biological tissues, rocks, semiconductors, nanomaterials, alloys, polymer films, etc. [1,2,3,4,5,6,7]. In the oil and gas industry, a precise digital twin of a sample is one of the cornerstones of Digital Rock workflow [8,9], which enables mathematical simulations of fluids flow in porous media, and estimations of the numerous physical and chemical characteristics of oil-bearing rocks. The advanced Digital Rock methodology consists of simulation on various scales including the nanoscale [10,11]. 

**Figure 2:**
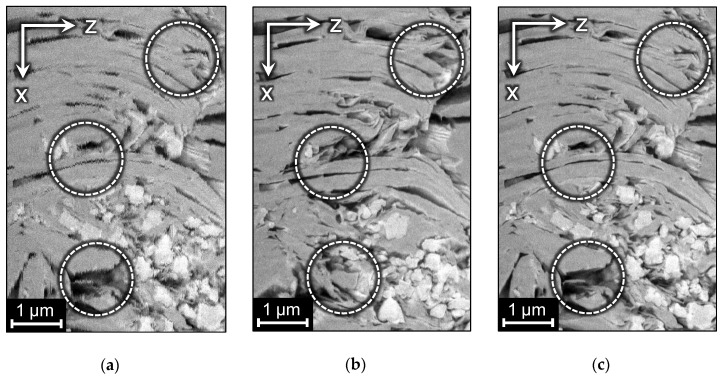
Distortions in the sample structure after alignment of slices and results of the proposed algorithm: (**a**) side view of the original 3D image; (**b**) side view of the image after conventional alignment; (**c**) side view of the image after the proposed alignment procedure.

FIB-SEM images have numerous peculiarities and specific artifacts, which prevent accurate segmentation and building a digital twin of a specimen. It means that the raw dataset of slices requires a lot of preprocessing steps before conducting a segmentation and any further numerical analysis. The important step is the alignment of the image stack along the Z-axis, because, in the general case, there are arbitrary geometrical distortions between adjacent slices. The severity of the distortions depends on the time of a frame scan and on experimental conditions, such as charge effects, mechanical and thermal instabilities [5]. For short scan time, distortions are random shifts mainly along the X and Y axes (see the designation of axes in Figure 1), whereas, for a long scan time, skew and rotation can additionally be present. Sometimes various parts of a slice are deformed in different ways. During focusing magnification can be changed; it leads to abrupt scaling transformation between slices. Certainly, researchers try to avoid distortions in the acquisition of images of samples, but it is not always possible. At least random shifts along the X and Y axes are almost unavoidable despite preliminary slice alignment by fiducial marks during scanning. Such displacements can be immediately revealed in the so-called side view when the stack is looked through not in original coordinate plane XY but in two other planes, XZ or YZ. Displacements of original slices look like shifted rows or columns and ragged edges in a side view (see Figure 2a).

Conventional algorithms used for the alignment of an image series [12,13,14,15] are based on the comparison of two adjacent slices. Due to local anisotropy in the orientation of edges in the XZ and YZ planes, existing alignment approaches make “smooth” distortions of pore space geometry additionally to the correction of ragged edges. The alignment algorithm tries to straighten the structures and make them perpendicular to the cutting plane, leading to unpredictable deformations in both the X and Y-directions along the Z-axis. Figure 2a,b demonstrate side views of a FIB-SEM image of a natural rock before and after alignment and outline the distortions.

Another reason for the distortions after an application of existing alignment methods consists in the combination of the experiment geometry and the so-called pore-back effect. In the case of porous media, the backside of pores is visible in the current slice whereas, in fact, it lies in the next layers. This not only makes segmentation a rather difficult task [16,17,18], but also influences the alignment procedure. If the algorithm focuses on the interiors of the pores, it will introduce a false bias in the Y-direction along the Z-axis, because the FIB and SEM columns are inclined to each other at the angle of 52 degrees.

A possible way to avoid these distortions is to scan a substantially bigger area so each frame of a FIB-SEM dataset would contain not only the region of interest, but also stable fiducial marks, which subsequently could be a reference for alignment. However, this approach is not usually used in practice as it decreases the resolution and increases overall scanning time.

Thus, reducing unreasonable transformations during alignment without fiducial marks still remains an important problem, because even small distortions in the pore space geometry are certainly capable of introducing errors into the permeability calculation. In this paper, we divide all the distortions into random real ones and “smooth” content-driven false deformations introduced by conventional alignment algorithms (as in Figure 2b). From the flow simulation point of view, the first group of distortions corresponds to an increase in the roughness on the surfaces and a decrease in the effective diameter of the filtration channels, which leads to a decrease in the permeability estimated. “Smooth” distortions also change the surface area and some other characteristics of pores. Precise pore space geometry and surface area of pores are extremely important for adequate simulation of physical and chemical processes.

In this paper, we propose an approach for the modification of conventional alignment procedures to avoid distortion of specimen geometry. Figure 2c demonstrates the outcome of our technique. The paper is organized as follows. In Section 2, we briefly consider algorithms usually used for alignment of FIB-SEM images. Section 3 describes the considered samples and images. Section 4 contains a description of the proposed method. The results are presented and discussed in Section 5. Finally, in Section 6, we conclude and outline future works.

## 2. Existing Approaches

The alignment of an image stack is based on the assumption of similarity of two adjacent slices, so algorithms iterate through the stack and find required transformations for each slice by the comparison with the previous one. We do not go deep into the description of alignment algorithms as there are many reviews on the subject [12,13,14,15], but rather concentrate on the methods used in software tools, such as Fiji [19,20] and Avizo^®^ [21].

Many of them exploit area- or intensity-based methods which, in contrast to feature-based algorithms, work with images without detecting salient objects [15]. They optimize some metrics that represent similarity of two images, such as the sum of absolute differences (SAD), mean squared error (MSE), correlation coefficient (CC), and mutual information (MI). MI methods are the leading technique in the registration of multimodal images, particularly in medicine [22,23]. Intensity-based approaches are implemented in several software products. In previous years, IMOD was a popular program for the alignment of image stacks [24]. It has two independent modules for automatic and manual alignment. Automatic alignment can rely on SAD or CC between adjacent slices. At the present time, Avizo^®^ Software (Thermo Fisher Scientific, Waltham, MA, USA) [21] is one of the commercial products that provides several ways of alignment for various images. There is a separate module for work with FIB-SEM datasets, which includes alignment based on the minimization of the sum of squared differences between two sequential slices. It is also possible to choose a mask for alignment. For medical images, there is an open-source toolbox for intensity-based image registration, Elastix [25,26], which includes several optimization methods, transformation models and metrics.

Nowadays researchers often use Fiji [19], a package of the open-source software ImageJ [20] with many plugins for image analysis [17,27,28]. The most mentioned in the publication modules for alignment of FIB-SEM images are Linear Stack Alignment with Scale-Invariant Feature Transform (SIFT) [29] and StackReg [30]. StackReg implements an iterative pyramidal approach for image registration and provides a finding of subpixel shifts [31]. At each iteration from low to high resolution, images are approximated with splines. Displacements between them are found by a non-linear method of least squares. Linear Stack Alignment with SIFT (previously JavaSIFT) exploits a feature-based method. It detects feature points in images by SIFT [32], and then applies RANSAC (RANdom SAmple Consensus) [33] for finding the matrix of transformation from one slice to another. The plugin has flexible settings and can log calculated transformation matrices.

Misaligned slices in a FIB-SEM image are similar to frames in a video with the camera shake effect. Therefore, an alignment procedure can rely on video stabilization techniques. For example, the Fiji plugin Image Stabilizer [34] implements the Lucas–Kanade method of calculation of optical flow [35].

Elastic Stack Alignment [36] is a part of the TrackEM2 Fiji module intended for non-linear registration of image series [37]. Each image is considered as a mesh of regular triangles with springs connecting their vertices. There are also zero-length springs between corresponding locations of two overlapping slices. This system relaxes to the state when the stack is deformed minimally. Elastic deformations can model any deformation that could occur in image data, but it does not produce a single transformation matrix for the whole image and there is a risk of local and large-scale distortions.

Concerning FIB-SEM images, authors usually consider alignment as an important preprocessing step, because it also influences the segmentation quality [17,38]. However, in most cases, an algorithm or software product is briefly mentioned, the quality of alignment is not evaluated numerically and only few authors try to prevent distortions in the sample structure. For example, in the paper [16], displacements are found by minimizing sum squared differences between images, accounting only for voxels below a certain threshold. It is supposed to guarantee that the alignment is computed based on the stable background structures, i.e., pore-backs. However, this is not a robust and universal approach. 

In paper [39], the authors note distortions introduced by traditional alignment methods and emphasize the importance of preserving the correct structure for subsequent work. They develop a method for alignment of FIB-SEM images of neurons which are supposed to be spherical, so the main idea is to find such corrections that lead to the expected shape of organelles. This approach cannot be applied to images of natural rocks because pores have arbitrary shape. 

The method presented in paper [38] is aimed at compensating pixel size variations using affine transformations. The first step is a pre-alignment procedure restricted to translations only. Then the pre-aligned dataset is smoothed along the z-axis with a median filter, creating a template to which the raw data is aligned using affine transformations. To avoid distortions, the authors propose to use template matching at the pre-alignment step, which relies on markings created at the surface of the sample. They work with biological materials and the top part of their first sample is a perfectly flat surface onto which the cells were growing, so the fiducial marks are straight lines orthogonal to the imaging plane. For the second sample, alignment marks are not available due to the specific preparation technique. In this case, the authors use the SIFT algorithm and note that it disturbs the global shape of objects across long distances. 

## 3. Datasets

**Figure 3:**
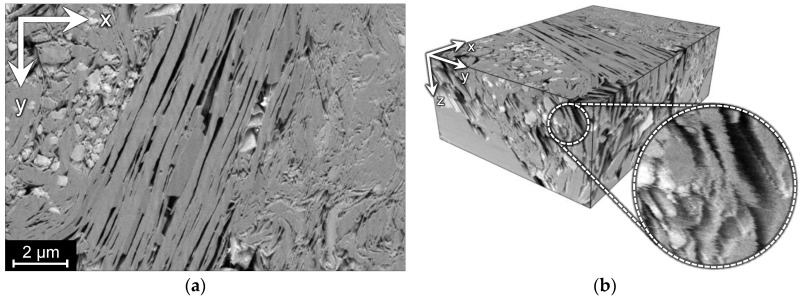
Image A: (**a**) a slice in the XY-plane; (**b**) 3D visualization and enlarged fragment of its side demonstrating misalignment in the XY-plane, which looks like shifted rows and ragged edges in the XZ and YZ side views.

We demonstrate the proposed method on two FIB-SEM images of tight sandstone and one synthetic dataset. Two FIB-SEM images were acquired with the FEI Helios 660 DualBeam™ microscope. We refer to these images as A and B. Image A contains 500 slices with the size of 1536 × 1024 pixels and the spatial resolution of 9.7 × 9.7 × 10 nm per voxel. Figure 3 shows a slice of image A and its 3D visualization with an indication of misaligned slices. While looking through the slices, we have noticed slight rotations and stretches in the image, so we assume more complex affine transformations here rather than displacements only. Ground truth is unavailable for this dataset, so the alignment can be assessed only qualitatively or by non-reference numerical metrics.

**Figure 4:**
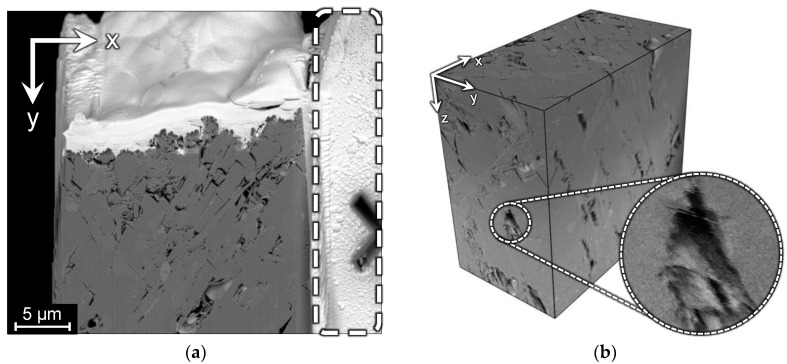
Image B: (**a**) a slice in the XY-plane including unchanged fragment outlined by the dashed line; (**b**) 3D visualization of the cropped region and enlarged fragment demonstrating misalignment in the XY-plane, which looks like shifted rows and ragged edges in the XZ and YZ side views.

Image B has 1010 slices with a size of 2048 × 1768 pixels, and the resolution is about 17 nm per voxel. This image was acquired with short scan time, and only displacements along the X and Y axes are present. In image B, there is a fragment of the sample, which is not affected by FIB and remains unchanged on all slices. Such a way of obtaining of FIB-SEM images is unusual, because the acquisition procedure commonly aims to provide higher resolution in the milled part of the sample. Figure 4a shows a slice of image B, where the unchanged fragment is outlined by a dashed line, and the 3D visualization with an indication of misaligned slices. There is a part of the fiducial mark that comprises half of the cross, which can be used for precise alignment. This alignment outcome can be considered as the ground truth (GT) for testing alignment methods based on the milled parts of slices.

**Figure 5:**
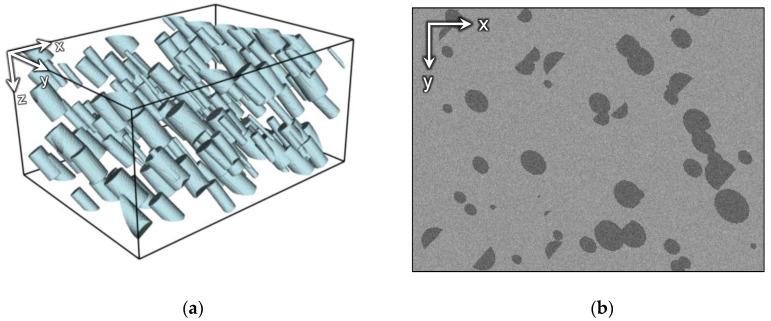
The synthetic image: (**a**) 3D visualization of the ground truth (GT); (**b**) a slice of the GT in the XY-plane.

**Figure 6:**
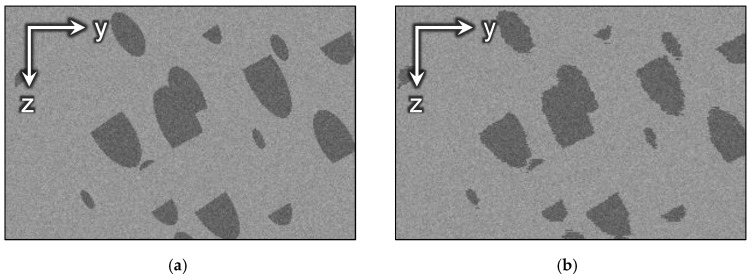
The side view of the synthetic image: (**a**) GT; (**b**) after displacement of slices.

We have also generated a synthetic dataset that imitates a sample with directed structures. The purpose is to demonstrate evidently how the existing alignment methods interfere with oriented edges and what changes they can bring into the initial structure of the sample. The dataset represents a simple geometry with homogeneous background and inclined cylinders, but any directed structures may be used. The image contains 200 slices with a size of 400 × 300 pixels. Additive white Gaussian noise was added to the images. To simulate the misalignment of FIB-SEM datasets, each slice is randomly displaced in the XY-plane. Figure 5 and Figure 6 demonstrate 3D visualizations of the ground truth and the cross-sections of the GT and the image with displaced slices.

## 4. Proposed Method

**Figure 7:**
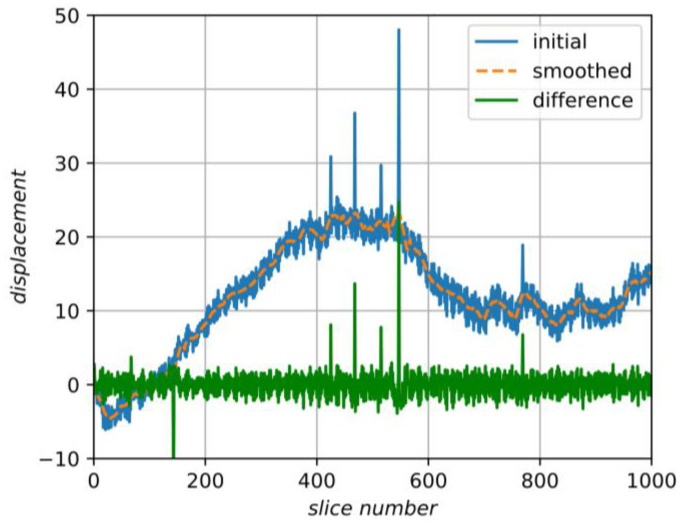
An example of smoothing of the initial signal and finding a high-frequency component.

For simplicity, we start an explanation of our method from the case of displacements only; after that, we generalize the technique to arbitrary affine distortions. We consider displacements produced by alignment algorithms as a signal depending on the slice number and containing two components. The first component corresponds to fluctuations in the data due to various random processes in the device. We assume that since these shifts are random, they correspond to a high frequency in the signal. The second component is caused by oriented structures or pores’ interiors, which should stay unaffected. They are supposed to be determinate over several slices, so the corresponding signal has a relatively low frequency. We suppose that this component can be found by smoothing the initial signal along slices and then it should be subtracted from the initial alignment transformations. Figure 7 shows an example of some initial signal, its smoothing and the difference between them. Thus, the main idea is to align one-by-one adjacent slices of the 3D image by some existing algorithm, then smooth the obtained transformations, subtract the smoothed component from the initial signal, and, finally, apply the result to the initial dataset.

In the general case, we consider not only translation, but an arbitrary affine transformation. This is an important issue when the time of a frame acquisition is long enough, so significant drift is accumulated during the scan of one slice. Operating with matrices of affine transformations, we use the notation M= (A | B), which means that the new coordinate $x^{\prime }=\;Ax+B$. Therefore, the composition of two transformations is equal to $M_{2}\times M_{1}=\;\left(A_{2}A_{1}\;|\;A_{2}B_{1}+B_{2}\right)$ and the inverse transformation is $M^{-1}=\;\left(A^{-1}\;|-A^{-1}B\right)$.

Assuming that the stack was aligned by one of the existing methods described in Section 2 (for example, minimizing SAD, JavaSIFT, Image Stabilizer, etc.), so, we have obtained affine transformation matrices $M_{i}$ for each slice *i*. According to the previous assumptions, there is a composition of two components $M_{i\;\mathrm{device}}$ and $M_{i\;\mathrm{structure}}$:

(1) $$M_{i}=\;M_{i\;\mathrm{device}}\times \;M_{i\;\mathrm{structure}}$$

Assuming that $M_{i\;\mathrm{structure}}$ can be found by smoothing of the initial transformations $M_{i}$ along slices, we can calculate $M_{i\;\mathrm{device}}$, which represents true displacements:

(2) $$M_{i\;\mathrm{device}}=M_{i}\times M_{\mathrm{smooth}\;i}{}^{-1}$$

To smooth an affine transformation, we do not work with its coefficients directly, but use decomposition into independent components, such as rotation, scaling, etc. There are many ways to decompose an affine transformation [40], but we have not found a visible difference in those ways for the given problem. We use a basis that consists of rotation R, scaling S, shear X and translation T:

(3) $$M_{i}=\left(S_{i}\;X_{i}\;R_{i}\;|\;T_{i}\right)$$

where $M=\left(\begin{array}{cc} a_{00} & a_{01}\\ a_{10} & a_{11} \end{array}|\begin{array}{c} \Delta x\\ \Delta y \end{array}\right)$, $S=\left(\begin{array}{cc} s_{x} & 0\\ 0 & s_{y} \end{array}\right)$, $X=\left(\begin{array}{cc} 1 & h_{x}\\ 0 & 1 \end{array}\right)$, $R=\left(\begin{array}{cc} \cos\alpha & \sin\alpha \\ -\sin\alpha & \cos\alpha \end{array}\right)$, $T=\left(\begin{array}{c} \Delta x\\ \Delta y \end{array}\right)$. Slice number i is omitted to simplify formulae, but, obviously, the coefficients are calculated for each slice independently.

Solving the system,

(4) $$\{\begin{array}{c} a_{00}=s_{x}\left(\cos\alpha -h_{x}\sin\alpha \right)\\ a_{01}=s_{x}\left(\sin\alpha +h_{x}\cos\alpha \right)\\ a_{10}=-s_{y}\sin\alpha \\ a_{11}=s_{y}\cos\alpha \end{array}$$

we find that in one special case $a_{11}=0$, then α=π/2 and $s_{y}=-a_{10}$. In other cases, denoting the $a_{00}a_{11}-\;a_{01}a_{10}$ as det(M):

(5) $$\{\begin{array}{c} \alpha =arctan\left(-a_{10}/a_{11}\right)\\ s_{y}=-a_{11}/\cos\alpha \\ s_{x}=\det\left(M\right)/s_{y}\\ h_{x}=\left(a_{00}a_{10}+a_{01}a_{11}\right)/\det\left(M\right) \end{array}$$

**Figure 8:**
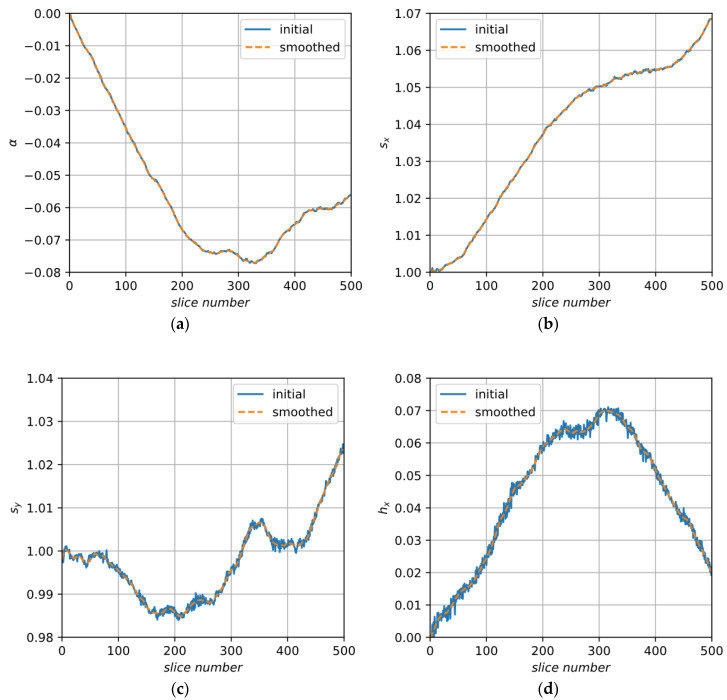
Initial and smoothed elements of decomposition for sample A: (**a**) angle α; (**b**) stretch factor along the X axis, $s_{x}$; (**c**) stretch factor along the Y axis, $s_{y}$; (**d**) factor of shear along the X axis, $h_{x}$.

**Figure 9:**
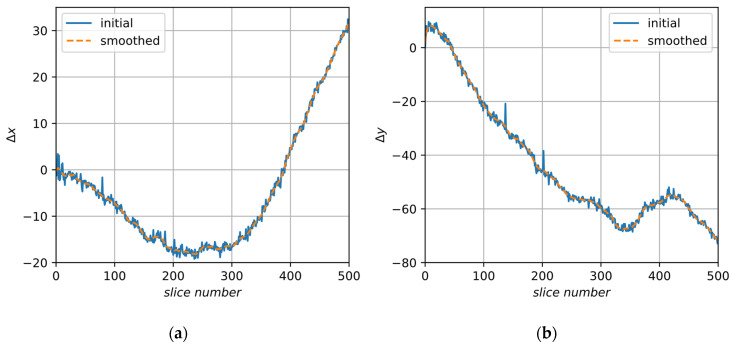
Initial and smoothed elements of decomposition for sample A: (**a**) displacement along the X axis, Δx; (**b**) displacement along the Y axis, Δy.

After decomposition, we have obtained vectors of independent coefficients for each slice. Now, coefficients can be smoothed along slices. Figure 8 and Figure 9 demonstrate the initial and smoothed signals of decomposition elements obtained for image A.

For smoothing, we use Savitzky–Golay filter [41], which fits successive sub-sets of adjacent data points with a low-degree polynomial by the method of linear least squares. From smoothed coefficients, new matrices $S_{\mathrm{smooth}\;i},\;X_{\mathrm{smooth}\;i},\;R_{\mathrm{smooth}\;i},\;T_{\mathrm{smooth}\;i}$ and, finally, $M_{\mathrm{smooth}\;i}$ are produced for each slice:

(6) $$M_{\mathrm{smooth}\;i}=\left(S_{\mathrm{smooth}\;i}\;X_{\mathrm{smooth}\;i}\;R_{\mathrm{smooth}\;i}\;|T_{\mathrm{smooth}\;i}\right)\;$$

**Figure 10:**
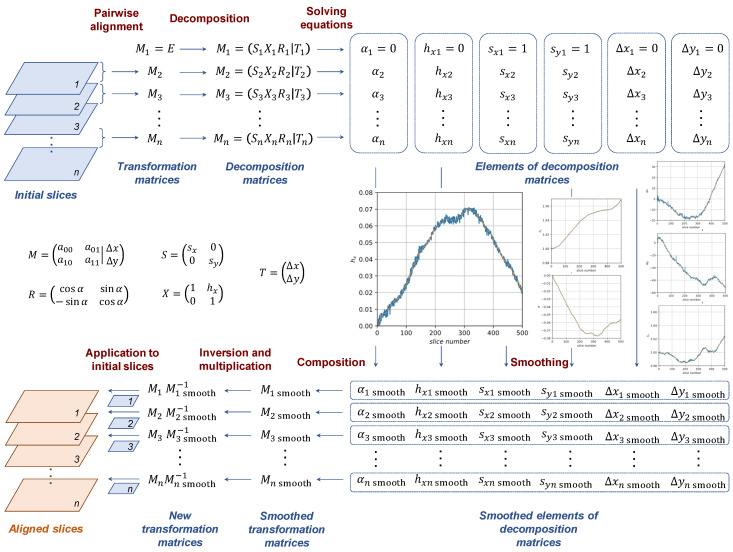
A scheme of the proposed algorithm.

The final step is the computation of inverse matrices $M_{\mathrm{smooth}\;i}^{-1}$ and then $M_{\mathrm{device}\;i}$ by formula (2). One can see the result of the application of $M_{\mathrm{device}\;i}$ to each slice in Figure 2c. Figure 10 illustrates the whole scheme of the proposed algorithm.

## 5. Results and Discussion

### 5.1. Quality Metrics for Alignment

In the case of available GT, that is, when the correct displacements are known, the obvious way to evaluate the quality of alignment is to calculate the arithmetical mean of differences between them and those found by the algorithm (Normalized Absolute Differences):

(7) $$NAD_{x}=\frac{1}{n}\overset{n}{\underset{i=1}{\sum }}\left|\Delta x_{true\;i}-\;\Delta x_{i}\right|$$

where n is total number of slices, $\Delta x_{i}$ are displacements along x-axis calculated by the algorithm for the $i^{th}$ slice, and $\Delta x_{true\;i}$ are true displacements along corresponding axes for the $i^{th}$ slice. The corresponding value for the Y axis is calculated in the same way.

However, an algorithm can make a mistake only in some small range of slices and, because of this, the rest of the slices can be biased from the correct position by the fixed value. In this case, the overall structure is preserved but the penalty of the alignment metrics (7) is unreasonably high. To avoid this, we introduce Normalized Absolute Differences of Gradients and use it in combination with NAD to evaluate alignment quality. This value is calculated according to the principle of differentiation and, therefore, ignores constant shifts:

(8) $$NADG_{x}=\frac{1}{n-1}\overset{n-1}{\underset{i=1}{\sum }}\left|\left(\Delta x_{true\;i+1}-\Delta x_{true\;i}\right)-\left(\Delta x_{i+1}-\Delta x_{i}\right)\right|$$

We also compute arithmetical means of the values for both axes $NAD_{mean}$ and $NADG_{mean}$ and use them as main criteria of alignment quality.

When there is no ground truth available, we can only estimate the visible roughness of edges in the image. We use the metrics presented in the paper [42]. The main idea is to detect and evaluate ragged edges between pores and solid matrix along the Z axis in the 3D image (see Figure 2a). The morphological operations tophat and bothat make it possible to find such regions, because tophat highlights small light details in the image and bothat, on the contrary, marks small dark regions.

(9) tophat(Im)=Im−opening(Im)

(10) bothat(Im)=closing(Im)−Im

where opening(Im) is morphological opening, closing(Im) is morphological closing. 

**Figure 11:**
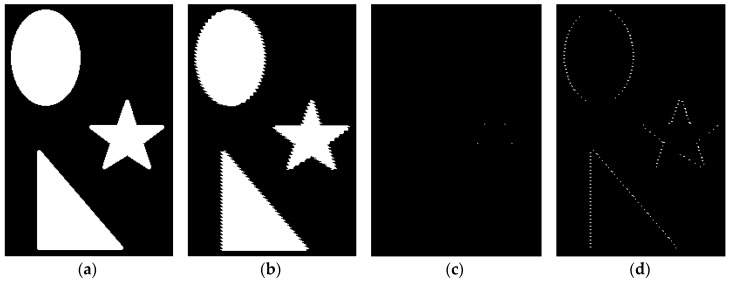
Application of bothat to a binary image: (**a**) initial image; (**b**) image with shifted rows; (**c**) bothat applied to the initial image; (**d**) bothat applied to the image with shifted rows.

Figure 11 demonstrates the application of bothat to the binary image and the same image with shifted rows. There are only solitary pixels in the case of smooth edges (Figure 11c), whereas shifted rows are detected and look like indented lines (Figure 11d). Tophat has a similar effect. 

This approach is easily generalized for 3D greyscale images. We use a 3-dimensional structure element which connects the central pixel with pixels along the Z axis, but not with pixels in the XY plane, because of misalignment resulting in ragged edges along the Z axis. We also apply thresholding to convert the result of tophat and bothat into a binary image and to make the metrics insensitive to noise. Therefore, Roughness Metrics can be introduced as:

(11) RM=sum( tophat(Im)>T or bothat(Im)>T )

We also calculate the Structure Similarity Index Measure SSIM [43] between the ground truth and aligned 3D images.

### 5.2. Alignment of the Synthetic Image

**Figure 12:**
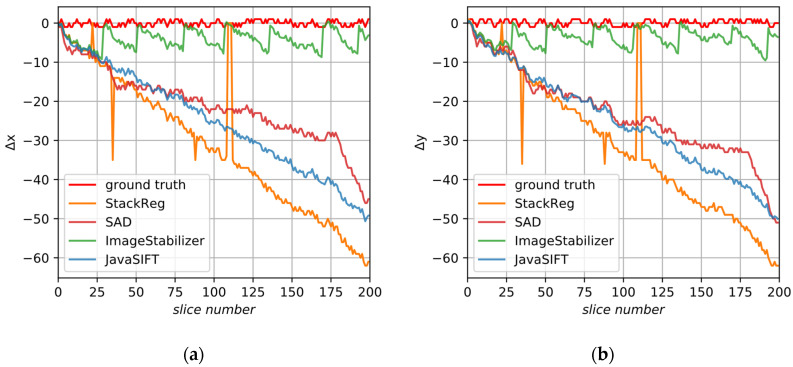
The displacements calculated by existing alignment algorithms for the synthetic sample: (**a**) in the X axis; (**b**) in the Y axis.

The synthetic sample clearly demonstrates “smooth” deformations from the alignment algorithms. We conducted alignment by comparison of two adjacent slices and minimization of the sum of absolute differences between their intensities. We have also tested Fiji plug-ins, namely Linear Stack Alignment with SIFT/JavaSIFT, StackReg, Image Stabilizer and Elastic, restricting alignment to parallel translations. Figure 12 demonstrates the ground truth displacements and the results of the applied alignment procedures, except the Elastic plugin, because it does not produce a sole transformation matrix for adjacent slices. There are sudden leaps in Image Stabilizer results, because, as we supposed, maximum displacements from the initial slice are limited. StackReg also makes similar mistakes because of the specific kind of image. SAD and JavaSIFT do not demonstrate breaks in the alignment but introduce the above-mentioned deformations of the image. 

**Figure 13:**
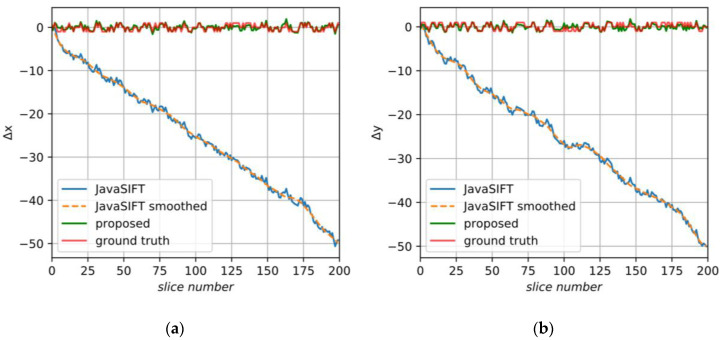
The displacements found by the proposed algorithm for the synthetic sample: (**a**) in the X axis; (**b**) in the Y axis.

We applied the correction procedure to the SAD and JavaSIFT results. Despite the fact that there were integer shifts in the initial dataset, it is a little better to use subpixel alignment as a basis for the proposed method. JavaSIFT calculates displacements with subpixel precision and has an option to interpolate the output image. Additionally, it is a ready and convenient tool which makes it possible to log transform matrices, so further we will rely on this plugin. Figure 13 shows the initial and smoothed JavaSIFT signals, ground truth data and, close to them, the final displacements of the proposed method.

**Figure 14:**
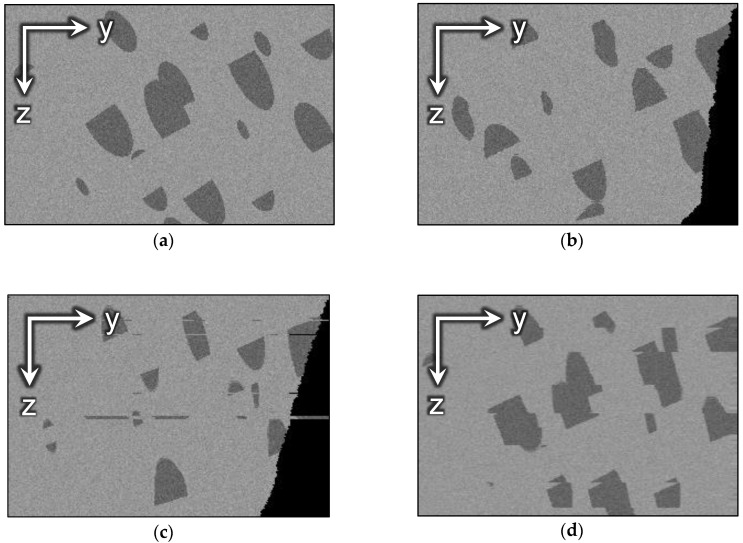

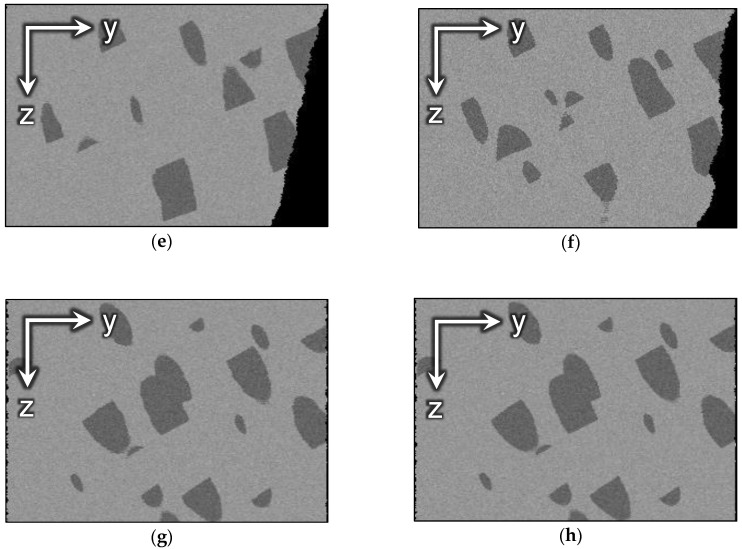
The side view of the synthetic sample: (**a**) GT; (**b**) aligned with SAD; (**c**) aligned with StackReg; (**d**) aligned with ImageStabilizer; (**e**) aligned with JavaSIFT; (**f**) aligned with Elastic; (**g**) aligned with the proposed algorithm based on SAD; (**h**) aligned with the proposed algorithm based on subpixel JavaSIFT.

Side views of the GT and aligned images are demonstrated in Figure 14. As one can see, the conventional methods have changed the 3D image in an unpredictable manner, resulting in a completely different sample structure, and only the proposed algorithm has preserved the initial geometry.

Alignment metrics are presented in Table 1: normalized absolute differences NAD for both axes separately and the average value, normalized absolute differences of gradients NADG and roughness metrics RM. Lower values mean better alignment, except SSIM. ImageStabilizer has low NAD metrics, but this is because it jumps to the initial state when it exceeds some limit. Both ImageStabilizer and StackReg have high RM because of disruptive results. Concerning the Elastic plugin, we are able to calculate only roughness metrics and visually estimate the alignment quality. SAD and JavaSIFT have low NADG metrics, but high NAD. The proposed algorithm has shown the best results for all metrics, except that JavaSIFT gives slightly lower roughness.

To illustrate the influence of precise alignment on the morphology of the sample, we calculate Minkowski functionals [44,45] for the synthetic image. Minkowski functionals are basic geometric measures defined for binary structures, so they require segmented images. For 3D images, the first functional V is just the total volume of pores, the second one S measures the total interfacial area between pore and solid, the third B corresponds to mean curvature of this interface, and the last one χ indirectly represents connectivity of pore space. Table 2 shows Minkowski functionals calculated for the initial synthetic image, drifted, aligned with JavaSIFT and aligned by the proposed procedure. As one can see, misalignment significantly changes the values of functionals, and, in particular, increases the interfacial area, which is a very important parameter for modelling physical and chemical processes. Alignment returns the functionals’ values closer to the initial dataset, and the results of the proposed method are slightly better than for existing tools. Nevertheless, in the assessment of alignment quality, we rely on the metrics $NAD_{mean}$ and $NADG_{mean}$. Minkowski functionals are an example that highlights the importance of alignment for restoring morphological characteristics.

### 5.3. Alignment of Image A

**Figure 15:**
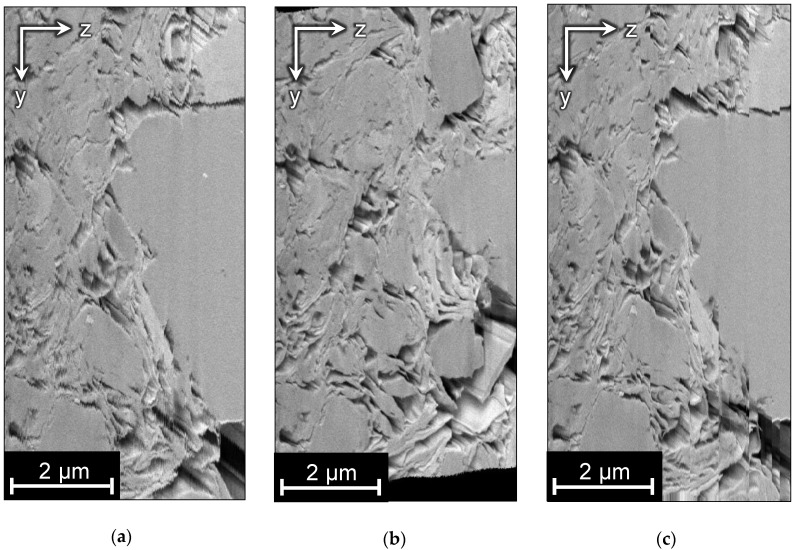

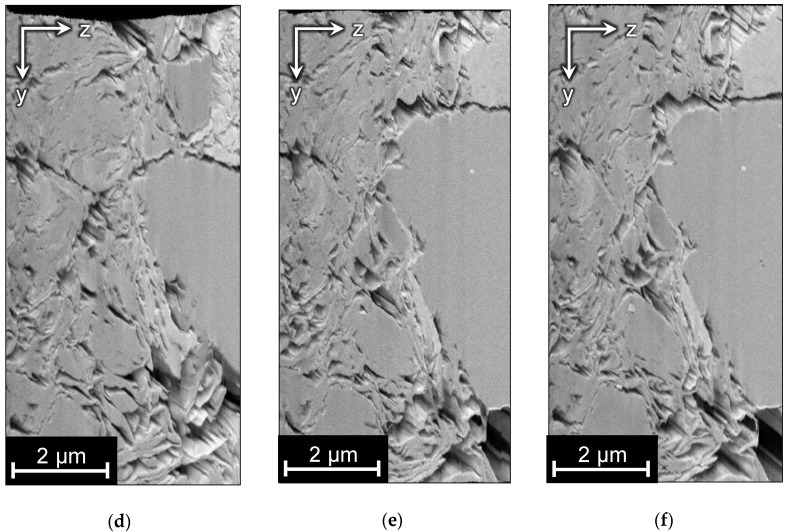
The side view for sample A: (**a**) the original image; (**b**) aligned with StackReg; (**c**) aligned with ImageStabilizer; (**d**) aligned with JavaSIFT; (**e**) aligned with Elastic; (**f**) aligned with the proposed algorithm.

Ground truth is unavailable for this dataset, so we suppose that the initial structure is correct except for random drift, and that alignment algorithms should not change it. As mentioned before, we assume arbitrary affine transformations for this image. Figure 15 shows the side views of the aligned images. StackReg and JavaSIFT change the geometry of the sample, and ImageStabilizer produces disruptive alignment. Elastic has acceptable results and does not distort the structure significantly. However, there are still ragged pore edges somewhere in the side views and, while looking through the stack, one can see slight relative movements and local distortions in the images.

The correction procedure for this sample was already described in Section 4 and illustrated in Figure 8. The resulting side view is shown in Figure 15f. By visual estimate, this alignment has better quality than others, because there are smooth edges and the structure is the closest to the initial one.

For this image, we can calculate only roughness metrics, which are presented in Table 3. JavaSIFT and the proposed algorithm have the best values, and ImageStabilizer the worst ones, as it gives disruptive results.

### 5.4. Alignment of Image B 

**Figure 16:**
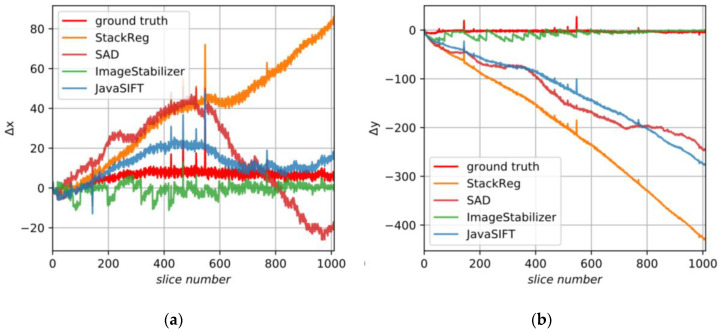
The displacements found by various algorithms for sample B: (**a**) in the X axis; (**b**) in the Y axis.

This image includes stable fiducial marks and, therefore, high-quality alignment can be done based on these marks. That alignment we consider as the GT. We assume only translations in this sample. Figure 16 demonstrates the GT and displacements found by the tested algorithms.

**Figure 17:**
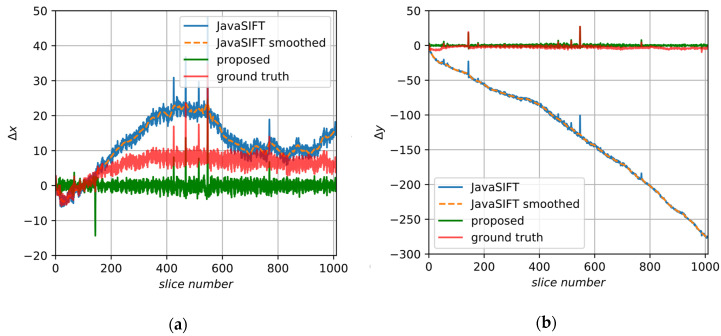
The displacements found by the proposed algorithm for image B: (**a**) in the X axis; (**b**) in the Y axis.

Figure 17 illustrates the proposed alignment procedure and shows the initial and smoothed JavaSIFT signals, ground truth data and final results. In this case, ground truth in the X axis does not fluctuate near zero but has a large-scale trend, which could occur because of various instabilities and operator’s actions during the experiment. This is not a very frequent situation, but, nevertheless, it induces mistakes because it disagrees with our initial assumptions. Our approach is aimed at removing any low-frequency trends from the initial signal and, without additional information, it cannot distinguish reasonable slow-altering components that should be a part of the final alignment from the false ones caused by sample anisotropy. We suppose that the additional information about correct low-frequency components can be found from logs of the experiment and metadata of the raw images.

**Figure 18:**
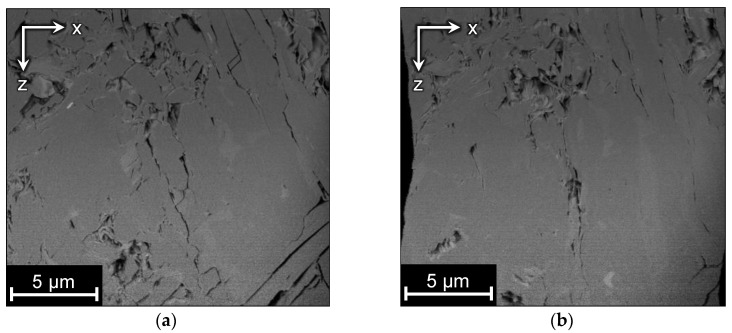

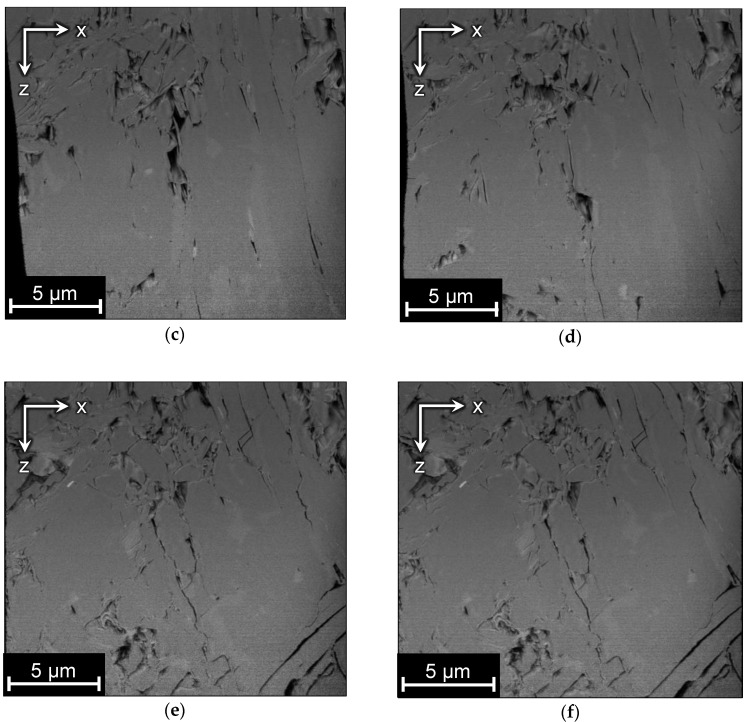
The side view of the image B: (**a**) GT; (**b**) aligned with SAD; (**c**) aligned with StackReg; (**d**) aligned with JavaSIFT; (**e**) aligned with Elastic; (**f**) aligned with the proposed algorithm.

Side views of aligned images are demonstrated in Figure 18. Only Elastic and the proposed algorithm have preserved the structure of the sample. 

Alignment metrics are presented in Table 4. Alignment metrics for image B. Again, the proposed approach has better values than other algorithms.

## 6. Conclusions

Conventional alignment algorithms introduce distortions in FIB-SEM images because they are confused by internal structures that are not oriented along the direction of the electron beam and pore-back effect in certain cases. A possible way to avoid these deformations is to scan not only the region of interest, but also stable fiducial marks so they are visible in each frame of a FIB-SEM dataset. However, this approach usually requires imaging a significantly bigger area and, therefore, decreases the resolution and increases the overall scanning time. Thus, reducing unreasonable transformations during alignment without fiducial marks still remains a relevant problem.

In this paper, we propose the method, which is aimed at extending existing alignment algorithms, and select from the produced transformation matrices only fast-altering components that correspond to distortions due to fluctuations in the device and represent real alignment transformation. We demonstrate the results on the synthetic data and one natural FIB-SEM image with known ground truth and measure the alignment quality by several proposed metrics. Our approach preserves the initial structure of the samples and demonstrates better alignment results in comparison with existing algorithms.

There are several limitations of the proposed method. First, it requires a sole transformation matrix for the whole slice. Second, it assumes that the input image has a valid structure so final transformations fluctuate along zero and do not have any constant shifts and low-frequency trends. In practice, it is not always taking place. In our future research, we are going to overcome these limitations by means of the looking for transformations for blocks of a slice and extraction of information about possible trends from metadata provided by the FIB-SEM system.

## Figures and Tables

**Table 1:** Alignment metrics for the synthetic sample.

Method	RM	$NAD_{x}\;$	$NAD_{y}\;$	$NAD_{mean}\;$	$NADG_{x}$	$NADG_{y}$	$NADG_{mean}$	SSIM
No alignment	1	0.51	0.55	0.53	0.60	0.51	0.56	0.43
SAD	0.19	21.1	23.3	22.2	0.23	0.26	0.24	0.28
StackReg	0.79	31.1	30.1	31.0	1.05	1.08	1.06	0.32
ImageStabilizer	0.30	4.08	3.82	3.95	0.53	0.51	0.52	0.53
JavaSIFT	0.08	25.0	25.4	25.2	0.28	0.28	0.28	0.43
Elastic	0.25							0.29
Proposed algorithm (SAD)	0.13	0.38	0.43	0.41	0.22	0.24	0.23	0.62
Proposed algorithm (subpixel JavaSIFT)	0.10	0.33	0.39	0.36	0.17	0.17	0.17	0.63

**Table 2:** Minkowski functionals for the synthetic sample.

Image	V	S	B	χ
GT	3,104,667	949,114	8933	−11
With displaced slices	3,104,667	1,077,930	10,159	−176
Aligned by JavaSIFT	3,104,667	986,102	9074	−62
Aligned by the proposed method	3,104,667	974,658	9368	−52

**Table 3:** Alignment metrics for image A.

Method	RM
No alignment	1.00
SAD	0.18
StackReg	0.05
ImageStabilizer	0.26
JavaSIFT	0.02
Elastic	0.10
Proposed algorithm	0.02

**Table 4:** Alignment metrics for image B.

Method	RM	$NAD_{x}\;$	$NAD_{y}\;$	$NAD_{mean}\;$	$NADG_{x}$	$NADG_{y}$	$NADG_{mean}$	SSIM
No alignment	1.00	6.30	2.87	4.59	2.69	1.57	2.13	0.31
SAD	0.77	17.9	130.4	74.2	0.42	0.65	0.54	0.27
StackReg	0.73	32.1	204.2	118.2	0.42	0.64	0.53	0.28
ImageStabilizer	0.62	6.88	5.83	6.35	0.45	0.78	0.62	0.34
JavaSIFT	0.64	7.98	59.4	33.7	0.51	0.74	0.62	0.31
Elastic	0.78							0.27
Proposed algorithm	0.64	6.27	2.74	4.51	0.38	0.48	0.43	0.35
